# Original and introduced lineages co-driving the persistence of *Brucella abortus* circulating in West Africa

**DOI:** 10.3389/fpubh.2023.1106361

**Published:** 2023-03-15

**Authors:** Zhiguo Liu, Miao Wang, Qi Shi, Xiaoping Dong, Liping Gao, Zhenjun Li

**Affiliations:** ^1^National Institute for Communicable Disease Control and Prevention, Chinese Center for Disease Control and Prevention, Beijing, China; ^2^Vocational and Technical College, Inner Mongolia Agricultural University, Baotou, China; ^3^Comprehensive Office, Ulanqab Center for Disease Control and Prevention, Jining, Inner Mongolia, China; ^4^National Institute for Viral Disease Control and Prevention, Chinese Center for Disease Control and Prevention, Beijing, China; ^5^Changzhi Medical College, Changzhi, Shanxi, China

**Keywords:** *Brucella abortus*, species/biovars, genetic diversity, MLST, MLVA, WGS-SNP

## Abstract

**Introduction:**

Brucellosis, a serious public health issue affecting animals and humans, is neglected in West Africa (WA).

**Methods:**

In the present study, bio-typing, multi-locus sequence typing (MLST), multiple-locus variable-number tandem repeat analysis (MLVA), and whole genome sequencing single-nucleotide polymorphism (WGS-SNP) analysis were used to characterize the *Brucella abortus (B. abortus*) strains from WA.

**Results:**

All of the 309 strains analyzed in this study were extracted and downloaded from the international MLVA bank and were from 10 hosts (cattle, humans, ovine, buffalo, dromedaries, horse, sheep, zebu, dog, and cat) distributed in 17 countries in WA. Based on the bio-typing, three biovars, dominated by *B. abortus* bv.3, were observed and reported across seven decades (1958–2019). With MLST, 129 *B. abortus* strains from the present study were sorted into 14 STs, with ST34 as the predicted founder. These 14 STs clustered into the global MLST data into three clone complexes (C I–C III) with the majority of strains clustering in C I, while C II forms an independent branch, and C III harbors three STs shared by different continents. These data revealed that most cases were caused by strains from native lineages. According to the MLVA-11 comparison, 309 strains were divided into 22 MLVA-11 genotypes, 15 of which were unique to WA and the remaining seven had a global distribution. MLVA-16 analysis showed that there were no epidemiological links among these strains. Based on the MLVA data, *B. abortus* strains from WA have high genetic diversity, and predominated genotypes were descended from a native lineage. While the MLVA-16 globally highlights that the dominant native and few introduced lineages (from Brazil, the USA, South Korea, Argentina, India, Italy, Portugal, the UK, Costa Rica, and China) co-driving the *B. abortus* ongoing prevalence in WA. The high-resolution SNP analysis implied the existence of introduced *B. abortus* lineages, which may be reasonably explained by the movement and trade of dominant hosts (cattle) and/or their products.

**Discussion:**

Our results indicated that *B. abortus* strains in WA consist of native and introduced strains that necessitate control such as vaccination, testing, slaughtering, and movement control by the relevant country authorities to reduce brucellosis in livestock.

## Introduction

Brucellosis, a widespread bacterial zoonotic disease that can cause considerable suffering in humans and massive economic losses in the animal industry, is caused by Gram-negative facultative intracellular bacteria of the genus *Brucella* (1–3). The genus includes 12 species, among which *Brucella melitensis, B. abortus*, and *B. suis* are the most important species and responsible for the majority of human and animal brucellosis cases (4). It has been reported that there are ~500,000 new cases of human infection annually, especially in low-income tropical countries (5). Cattle are natural hosts of the intracellular pathogen *B. abortus*, which can also be isolated in other hosts, such as sheep, camels, and horses, and imposes a significant burden on the health and reproduction of these important livestock (6, 7). Infected animals can have live offspring following the initial abortion, and they may continue to shed the bacteria (8). Transmission to human occurs through unprotected handling of tissues or body fluids from infected animals, consumption of unpasteurized milk and milk products, or inhalation of *Brucella*-contaminated aerosols (9, 10). Although *B. abortus* causes less severe infections than *B. melitensis* (11), back pain and arthralgia are common symptoms in patients infected with *B. abortus* in Turkey (12).

A previous study reported that the annual economic burden of bovine brucellosis in Latin America is ~$600 million (13). In 2015, in Kazakhstan, around $21 million was spent on compensation for slaughtered animals with brucellosis, and an additional $24 million was spent on testing and screening of animals (14). Therefore, brucellosis can not only lead to substantial economic losses in the animal industry but also pose an ongoing threat to public health.

Countries declared brucellosis-free are located in Europe and Oceania, whereas the prevalence is high in enzootic countries in central and South America, Africa, and parts of Asia (15). In these developing countries, especially in Africa, livestock husbandry development is continuously challenged by brucellosis (16, 17). Bovine brucellosis remains the most widespread disease in animals and the main concern in sub-Saharan countries (18). In African countries, *B. abortus* has been reported in Sudan, in both cattle and their handlers (19), as well as in Gambia, Mali, Niger, and more frequently in Nigeria, Senegal, and Ivory Coast (20). Therefore, a comprehensive analysis of the genetic diversity of *B. abortus* strains and the epidemiology of the disease in animals and humans in sub-Saharan countries is necessary.

Molecular characterization of the predominated circulating strains is critical to understanding *B. abortus* diversity and epidemiology in the country. Multi-locus sequence typing (MLST) is a reliable tool for the characterization of *Brucella spp*. populations and the determination of phylogenetic relationships (21, 22); however, this method yields less detailed typing results than MLVA and WGS-SNP due to the lower-resolution power (21, 23). Multiple locus variable-number tandem repeat analysis (MLVA) enables *Brucella* genotyping to infer genetic diversity and investigate the geographic clustering of the isolates (24–26). Moreover, whole genome sequencing single-nucleotide polymorphism (WGS-SNP) has higher discriminatory power to efficiently track the origin and spread of *Brucella* strains, leading to be targeted and effective control of disease spread (27, 28). At present, MLVA has been used to explore the diversity of *B. abortus* biovar 3 isolated in West Africa (29) and investigate the epidemiological links of the *B. melitensis* in Egypt (30). Moreover, studies that used MLVA and WGS-SNP (31, 32) indicated that WGS-SNP analysis allows a better differentiation than MLVA-16. WGS-SNP analysis has been used to type, discriminate and track outbreak strains (31–33). Therefore, the purpose of this study was to use a series of molecular typing assays, including MLST, MLVA, and WGS-SNP, to investigate the species population, genetic diversity, geographical origin, and molecular epidemiology of *B. abortus* strains from West Africa (WA), to provide insight into the comprehensive understanding of the *B. abortus* brucellosis epidemiology features in WA, and to facilitate surveillance and control strategy development.

## Methods

### Source of MLST and MLVA genotyping data

The MLST genotyping data of 129 *B. abortus* strains (Table 1; Supplementary Table S1) were downloaded from the PubMLST (https://pubmlst.org/organisms/brucella-spp), including the key (code) of isolates, sequence type (ST), allotype of nine loci (aroA/cobQ/dnaK/gap/glk/gyrB/int_hyp/omp25/typE), *Brucella* species/biovars, host, country, continent, and years isolated. Moreover, the MLVA-16 genotyping data of 309 *B. abortus* strains (Table 2; Supplementary Table S2) from 17 WA countries, including Senegal, Egypt, Togo, Nigeria, Rwanda, Kenya, Guinea-Bissau, Uganda, Niger, Zimbabwe, Cameroon, Sudan, Tanzania, Guinea, Ivory Coast, Chad, and Gambia, were abstracted from the MLVA bank database (https://microbesgenotyping.i2bc.paris-saclay.fr/databases). The key (code) of strains, number of tandem repeats at each of the MLVA-16 loci, MLVA-8 genotype, MLVA-11 genotype, hosts, species/biovars, isolated regions, years isolated, and lineage groups were obtained. The MLVA-8 genotype, MLVA-11 genotype, and lineage group data from 19 *B. abortus* MLVA-8 and 21 MLVA-11 genotypes were not previously identified genotypes in the international MLVA bank database.

**Table 1:** Country, hosts, number of strains and sequencing type (ST) of 129 *Brucella abortus* strains from Africa used for multi-locus sequence typing (MLST) genotyping.

**Country**	**Host**	**Strains number**	**ST**
Cameroon	Bovine	1	32
Chad	Cattle, Bovine	8	2, 32, 34
Egypt	Cattle, Buffalo	11	1, 2
Ethiopia	Cattle	30	72
Kenya	Unknown	4	32, 38
Mozambique	Bovine, Cattle	8	1, 37, 72
Niger	Bovine	1	83
Nigeria	Unknown	7	34, 36
Rwanda	Human	1	32
Senegal	Bovine, Cattle	6	36, 82, 84
Sudan	Bovine, Cattle, Camel	32	2, 6, 33, 34
Tanzania	Human	1	32
Togo	Bovine	1	36
Uganda	Human	4	2, 6
Zambia	Bovine	2	1
Zimbabwe	Bovine, Cattle	12	1, 5, 6

**Table 2:** Country, MLVA-8, MLVA-11, host, species/biovar, number, and year of 309 *B. abortus* strains used for MLVA genotyping.

**Country**	**MLVA8**	**MLVA11**	**Host**	**Species biovar**	**Number**	**Year**	**Group**
Chad	34, 35, new type (nt8-8)	62, 63, nt11-13	Cattle	*B. abortus* bv3, bv6	3	Unknown	Abortus B
Cameroon	34	63	Cattle, Zebu	*B. abortus* bv3	3	1989–1990	Abortus B
Sudan	34	63	Dromedary	*B. abortus* bv3	3	1993	Abortus B
Togo	34	64, nt11-12	Cattle	*B. abortus* bv3	31	1979–1980	Abortus B
Uganda	34	64	Human	*B. abortus* bv3	5	1958	Abortus B
Guinea	34	317	Cattle	*B. abortus* bv3	2	1982–1992	Abortus B
Guinea-Bissau	34	317	Cattle	*B. abortus* bv3	6	1982	Abortus B
Kenya	34	317	Cattle	*B. abortus* bv3	10	2009	Abortus B
Rwanda	34	317	Cattle, Human	*B. abortus* bv3	14	1982–1983	Abortus B
Ivory coast	34	317	Cattle	*B. abortus* bv3	1	2009	Unknown
Gambia	34	317	Cattle	*B. abortus* bv3	1	Unknown	Unknown
Senegal	34, 137, 139, 142	63, 64, 310, 311, 312, 314, 315, 316, 317	Cattle	*B. abortus* bv1, bv3	138	1976–1979	Abortus B
Nigeria	28, 34, 35, new types 7 (nt8-2, 3, 4, 5, 6, 7, 9)	62, 63, 310, new types 11 (nt11-3, 4, 5, 6, 7, 8, 9, 10, 11, 14, 15)	Cattle, Sheep, Horse	*B. abortus* bv3	19	1976–2012	Unknown
Egypt	27, 28, 33	76, 79, 81, 82, 83, 400	Ovine, Buffalo, Cattle, human, dog, cat, and vaccine (*n* = 2)	*B. abortus* bv1	56	2002–2020	Abortus C, RB51_2308
Zimbabwe	28, 31, 37	74, 77, 82	Cattle	*B. abortus* bv1	4	Unknown	Abortus C
Tanzania	nt8-1	nt11-1, nt11-2	Cattle	*B. abortus* bv3	3	2012–2013	Unknown
Niger	134	313	Cattle	*B. abortus* bv3	4	1983	Abortus B
Africa	40, 178	66, 348	Cattle	*B. abortus* bv6	6	1959	Abortus C

### Analysis and visualization of MLST and MLVA data

All data in this study were cleaned and processed using Excel 2016 software (Microsoft Corporation, Redmond, WA, USA). For MLST analysis, genetic similarities between the STs of 129 strains from WA and 631 strains from other continents (PUBMLST) (Supplementary Table S1) were investigated using eBURST software 2.0. Finally, the genetic relationships between the isolates and allelic profiles of MLST data were analyzed with the software PHYLOViZ version 2.0 using the goeBURST Full Minimum Spanning Tree algorithms (34). Subsequently, MLVA data were processed according to genotype information, species/biovars, hosts, location, and years isolated for all *B. abortus* strains. A minimum spanning tree (MST) of *B. abortus* strains was constructed using BioNumerics 8.0 software based on the 309 *B. abortus* strains' MLVA-11 data (Supplementary Table S2) to investigate the host lineage, genotype distribution, and the geographical origin of the *B. abortus* strains. At the global level, an MST was constructed using the MLVA-11 data of 1,746 strains (Supplementary Table S2) to explore the geographical origin feature of genotypes from this study at the global level. Moreover, the MST based on the MLVA-16 data of 1,746 *B. abortus* strains (Supplementary Table S2) was used to determinate the relationship among strains at a global scale. In this study, the MST trees were weighted according to MLVA panels, as described by previous reports (35).

### Phylogenetic analysis based on the SNPs of 80 strains at the global level

WGS-SNP analysis was performed as previously described (36). Briefly, genomic alignment between the sample genome and reference genome (*B. abortus* 2308; Assembly ID: GCA_000054005.1) was performed using the MUMmer (37) and LASTZ (38) tools. The phylogenetic tree of 18 *B. abortus* strains from WA and 62 strains from outside WA (Supplementary Table S3) in GenBank was constructed using TreeBeST (39) based on the Maximum-Likelihood Phylogenies (PHYML) algorithm with 1,000 bootstrap replicates to investigate the genetic relationships of *B. abortus* strains.

## Results

### Species/biovars, host, and geographic distribution of 309 *B. abortus* strains

**Figure 1:**
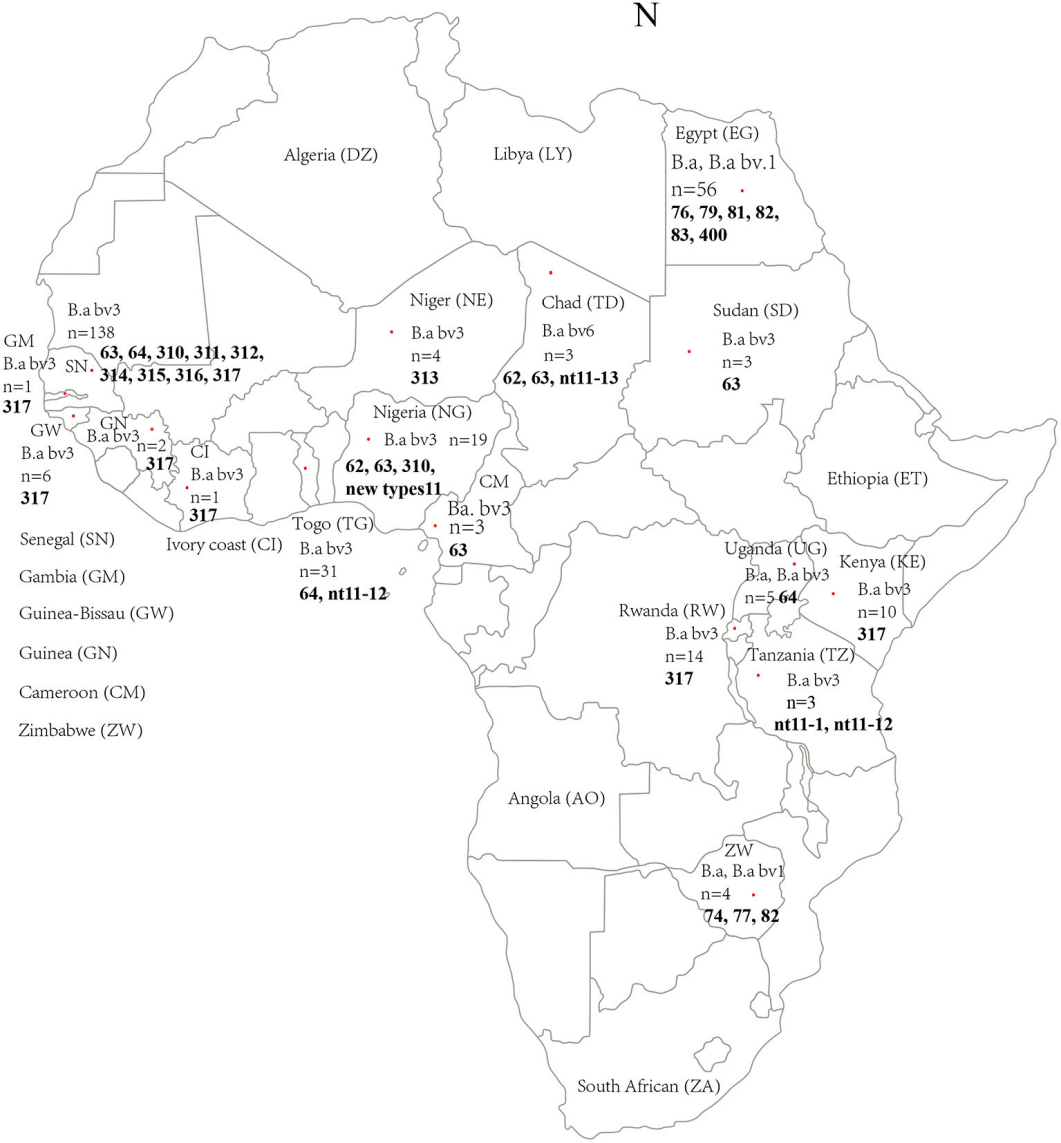
Geographic and genotype distribution features of 309 *B. abortus* in the present study. Red dot indicates the location (country) of the strains collected in this study. The letter in brackets is the abbreviation of the national name in the figure; *n*, Number of strains collected; figures marked with the bold refer into the MLVA-11 genotypes of strains from the location. The map of Africa used in this study was obtained from Google Maps (https://www.google.com/maps).

Of the identified biovars of 309 *B. abortus* African strains, 239 *B. abortus* bv 3, 9 *B. abortus* bv 1 and 6 *B. abortus* bv 6 were identified with 55 not typed to biovar level (Figure 1; Table 2). The *B. abortus* bv. 1 was distributed in Egypt (*n* = 6), Zimbabwe (*n* = 2), and Senegal (*n* = 1). *B. abortus* bv. 6 was found in Chad (*n* = 2), and the location of the remaining six is unknown. However, the *B. abortus* bv. 3 strains were distributed in 15 countries, i.e., all 17 countries except Egypt and Zimbabwe (Figure 1; Table 2). Based on the host, 280 strains were found in cattle, 8 in humans, 4 in ovine, 4 in buffalo, 3 in the dromedary, 2 in horse, 2 in sheep, 2 in zebu, 1 in a dog, and 1 in a cat, and the remaining two were vaccine strains (Table 2). A total of 309 *B. abortus* were collected from 17 countries, including 138 in Senegal, 56 in Egypt, 31 in Togo, 19 in Nigeria, 14 in Rwanda, and remaining countries contained 1–10 different numbers strains (Figure 1; Table 2). According to the MLVA data, strains were divided into the abortus B group (*n* = 209), the abortus C group (*n* = 55), and the abortus C_RB51_2308 group from Egypt (*n* = 11), and the remaining 24 strains were unknown. These strains were isolated between 1958 and 2020, including 5 in the 1950s, 147 in the 1970s, 43 in the 1980s, 7 in the 1990s, 13 in the 2000s, 46 in the 2010s, and 2 in 2020, and the year of isolation of the remaining isolates is unknown (Table 2; Supplementary Table S2).

### MLST genotyping characteristic of *B. abortus* strains in the present study

**Figure 2:**
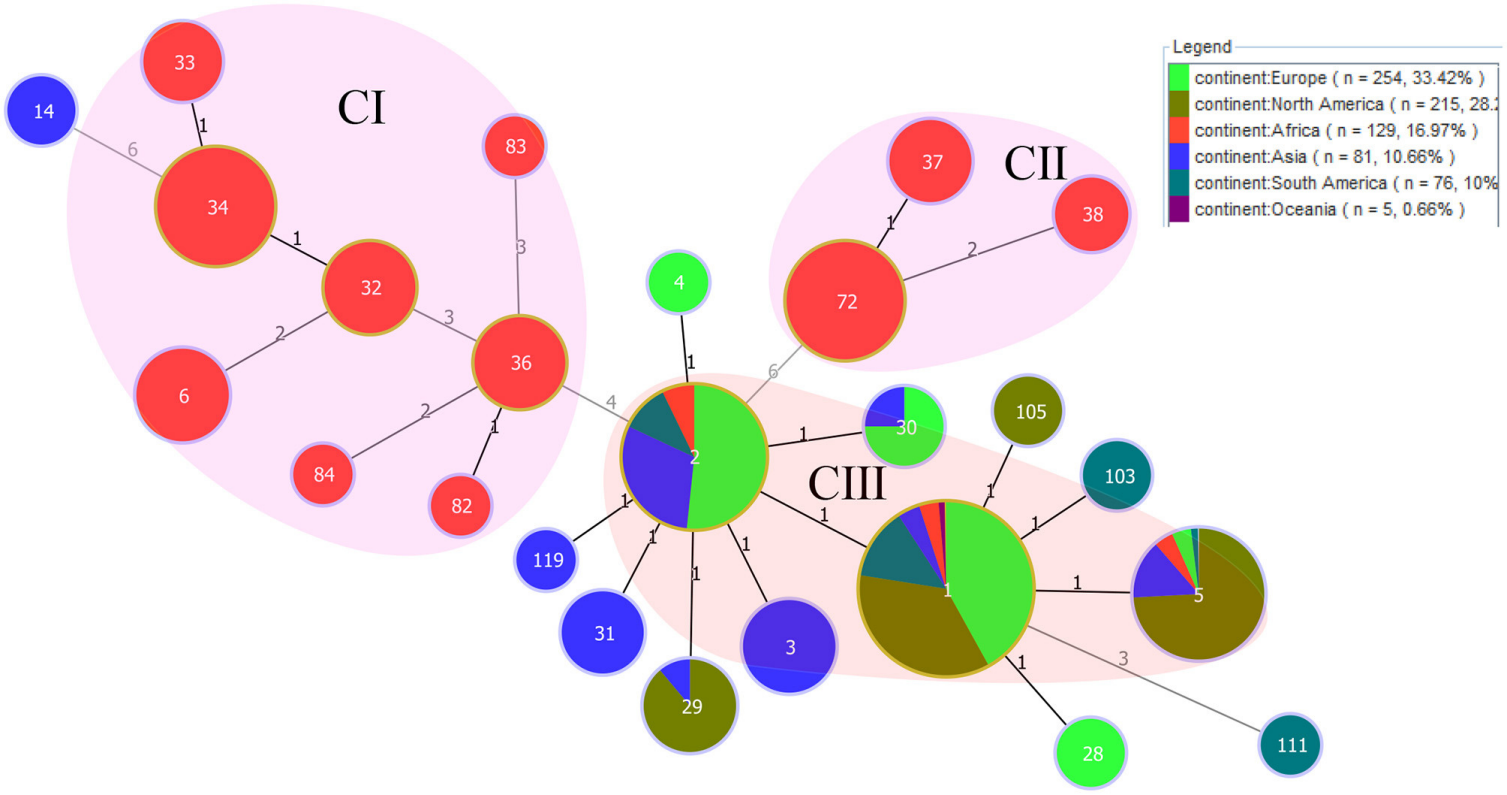
PHYLOViZ analysis showing the genetic relationships among STs of 760 *B. abortus* strains. Color coding according to continents. Circle size indicates the number of strains. Each circle represents a distinct ST, and the STs from the present study are shaded in pink **(C I, C II)** and light red **(C III)**.

The results of eBURST analysis showed that the 129 *B. abortus* strains from the present study could be sorted into 14 STs, including ST1 (*n* = 16), ST2 (*n* = 8), ST5 (*n* = 3), ST6 (*n* = 9), ST32 (*n* = 8), ST33 (*n* = 4), ST34 (*n* = 31), ST36 (*n* = 8), ST37 (*n* = 5), ST38 (*n* = 3), ST72 (*n* = 31), ST82 (*n* = 1), ST83 (*n* = 1), and ST84 (*n* = 1) (Table 1). Of these, ST34 was the predicted founder and included two single locus variants, zero double locus variants, one triple locus variant, and 10 more distantly related (satellites) (Supplementary Figure S1). The ST34 is comprised of strains from three countries, including Sudan (*n* = 24), Nigeria (*n* = 4), and Chad (*n* = 3). The MST at the global scale indicated that among those strains (*n* = 760) from six continents, 14 STs in the present study were divided into three clone complexes (C I–C III) (Figure 2), of which C I and C II comprised strains from the present study and formed the independent complex. C I comprised eight STs (ST6, 32, 33, 34, 36, 82, 83, and 84; and strains from Cameroon, Chad, Kenya, Niger, Nigeria, Rwanda, Senegal, Sudan, Tanzania, Togo, Uganda and Zimbabwe) and C II contained three STs (ST37, 38, and 72; and strains from Ethiopia, Kenya, and Mozambique) (Figure 2). C III harbored three single STs (ST 1, 2 and 5; and strains from Chad, Egypt, Mozambique, Sudan, Uganda, Zambia, and Zimbabwe) that were shared between three and five continents: ST1 was shared by strains from Asia, Europe, North America, Oceania, and South America (Figure 2); ST2 was shared by strains from Asia, Europe, and South America; and ST5 was shared by strains from four continents, including Asia, Europe, North America, and South America (Figure 2).

### Diversity characteristics of MLVA-8 and MLVA-11 data of strains from this study

**Figure 3:**
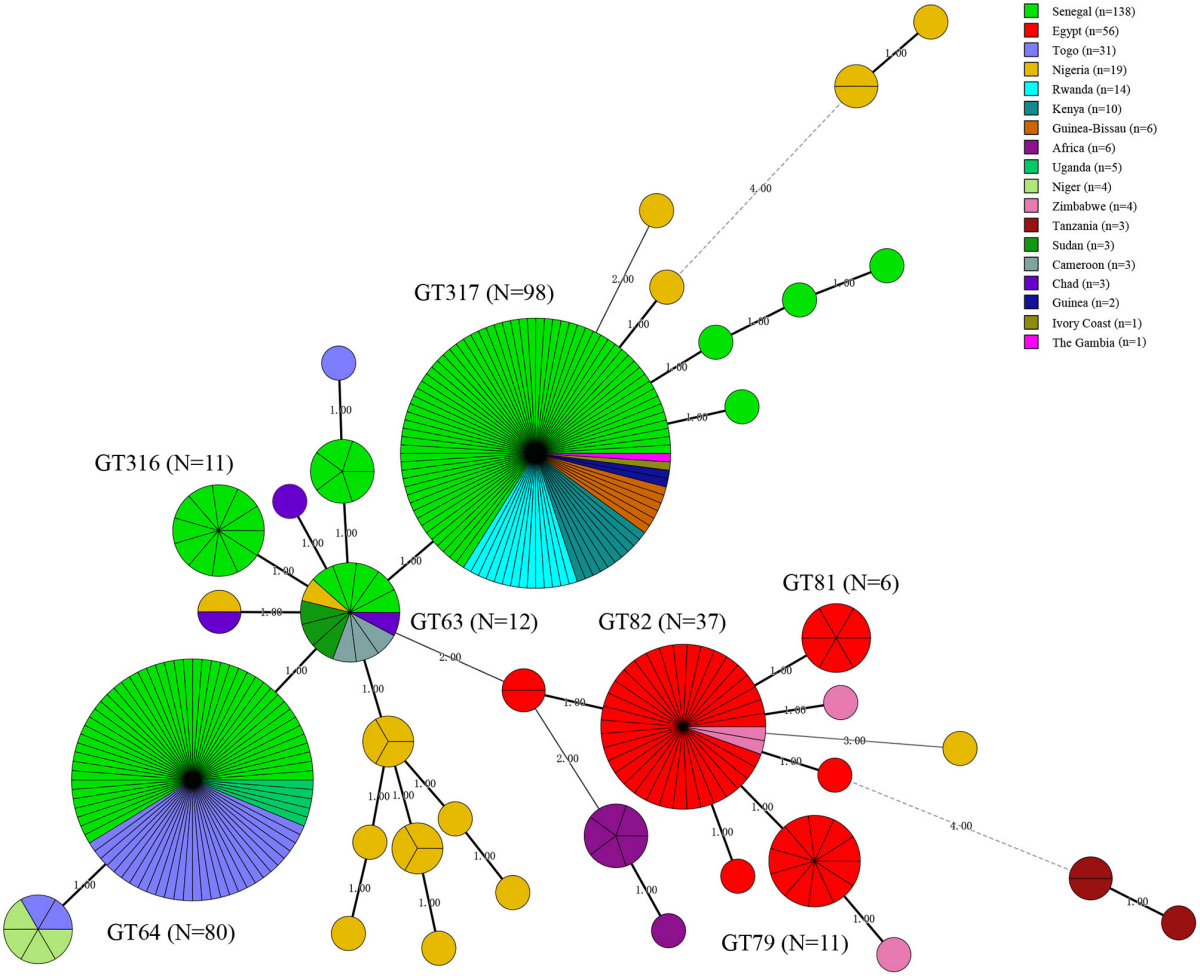
Minimal spanning tree (MST) of MLVA-11 genotype geographic distribution based on the multi-loci variable number of tandem repeats (VNTR) 11 data from West African *Brucella abortus* strains. Color coding according to countries distributed of MLVA-11 genotypes (the detail genotype profile is presented in Supplementary Figure S2), the predominated MLVA-11 genotypes in the present study were marked with the bold in figures. Circle size indicates the number of strains, GT indicates MLVA-11 Genotype. The number on the horizontal line is the number of site differences between genotypes.

A total of 13 known MLVA-8 genotypes and nine new genotypes (nt8-1–nt8-9) were recorded among 309 strains, known 13 MLVA-8 genotypes including 27 (*n* = 11), 28 (*n* = 45), 31 (*n* = 1), 33 (*n* = 2), 34 (*n* = 206), 35 (*n* = 1), 37 (*n* = 1), 40 (*n* = 5), 134 (*n* = 3), 137 (*n* = 1), 139 (*n* = 1), 142 (*n* = 2), and 178 (*n* = 1) (Table 2). According to the MLVA-11 analysis showed that a total of 37 MLVA-11 genotypes identified among 309 strains, and 22 out of 37 MLVA-11 genotypes were previously identified including 62 (*n* = 1), 63 (*n* = 12), 64 (*n* = 80), 66 (*n* = 5), 74 (*n* = 1), 76 (*n* = 2), 77 (*n* = 1), 79 (*n* = 11), 81 (*n* = 6), 82 (*n* = 37), 83 (*n* = 1), 310 (*n* = 1), 311 (*n* = 1), 312 (*n* = 1), 313 (*n* = 3), 314 (*n* = 1), 315 (*n* = 5), 316 (*n* = 11), 317 (*n* = 98), 348 (*n* = 1), and 400 (*n* = 1) (Table 2); and the remaining 15 new MLVA genotypes were first found in this study, then named them as into nt11-1–nt11-15 (Table 2). MLVA-11 genotype 317 (*n* = 98) and 64 (*n* = 80) are the predominant circulating lineages (Figure 3). Moreover, MLVA-11 genotypes 317 and 64 were mainly distributed in Senegal, and MLVA-11 genotype 82 was mainly distributed in Egypt. MLVA-11 genotype 63 was observed in Senegal, Uganda, and Cameroon (Figure 3).

### Geographic origin and genetic relatedness of *B. abortus* strains on a global scale

**Figure 4:**
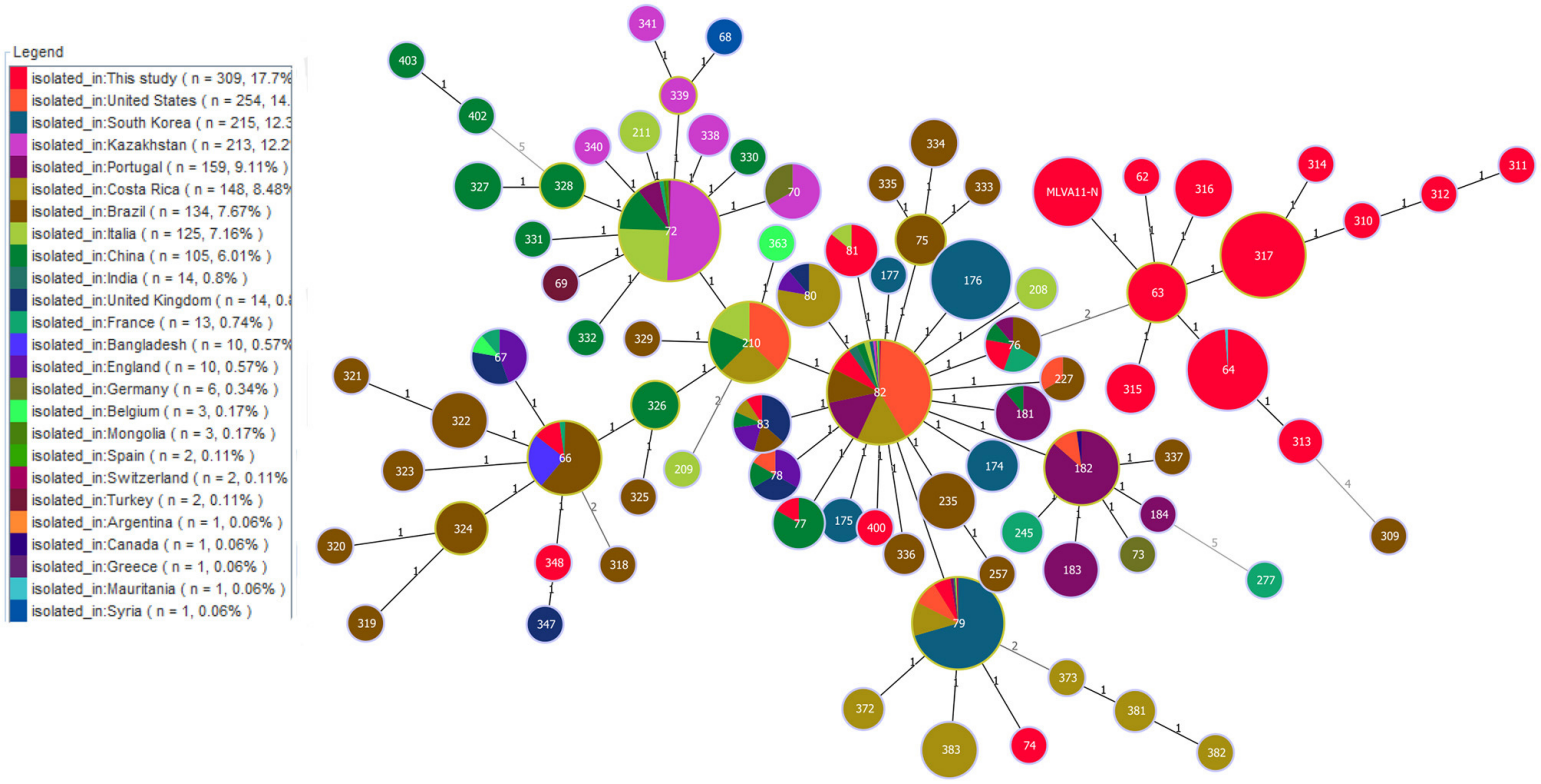
Geographic origin analysis of *B. abortus* strains in West Africa on a global scale based on the multi-loci variable number of tandem repeats (VNTR) 11 data worldwide. Color coding according to countries. Circle size indicates the number of strains.

Phylogenetic analysis of MLVA-11 data on *B. abortus* strains on a global scale showed that out of 22 MLVA-11 genotypes, 15 formed an independence lineage (Figure 4). Of these 15, except for three genotypes (MLVA-11 genotypes 74, 348, and 400) that were related to strains from other countries, the remaining 12 MLVA-11 genotypes formed a unique cluster (Figure 4). Moreover, strains from seven other MLVA-11 genotypes (MLVA-11 genotypes 66, 76, 77, 79, 81, 82, and 83) were shared with strains from 2 to 14 different countries (Figure 4). Of these, MLVA-11 genotype 66 was shared by strains from three countries, including Bangladesh, Brazil, and France; MLVA-11 genotype 76 was shared with strains from four countries, including Brazil, China, France, and Portugal; MLVA-11 genotype 76 was shared by strains from the present study and China; MLVA-11 genotype 79 was shared by strains from Costa Rica, India, Italia, Portugal, South Korea, Switzerland, and the USA (Figure 4); MLVA-11 genotype 81 was shared by strains from Italy and this study; MLVA-11 genotype 82 was shared by strains from Argentina, Belgium, Brazil, China, Costa Rica, France, Germany, India, Kazakhstan, Portugal, South Korea, Switzerland, the UK, and the USA (Figure 4); MLVA-11 genotype 83 was shared by strains from Brazil, China, Costa Rica, England, and the UK (Figure 4).

### Molecular epidemiological links of *B. abortus* in West Africa and on a global scale

**Figure 5:**
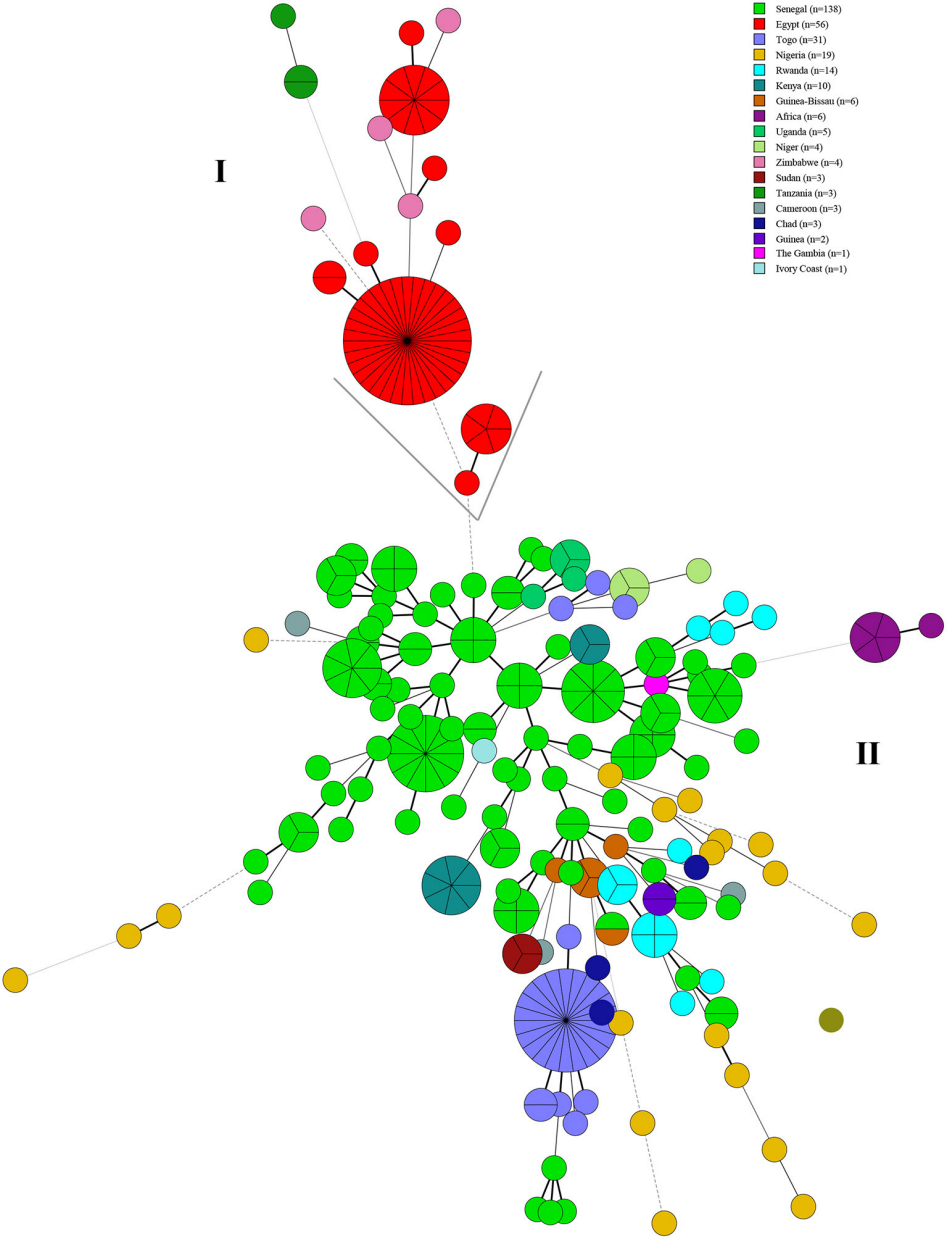
Molecular epidemiology relatedness of *B. abortus* strains from West Africa based on the MLVA-16 data of 309 *B. abortus*. Color coding according to the country in this study. Circle size indicates the number of strains. Branch **(I)** comprised strains from Egypt, Zimbabwe, and Uganda and branch **(II)** contained strains from the other 14 countries.

**Figure 6:**
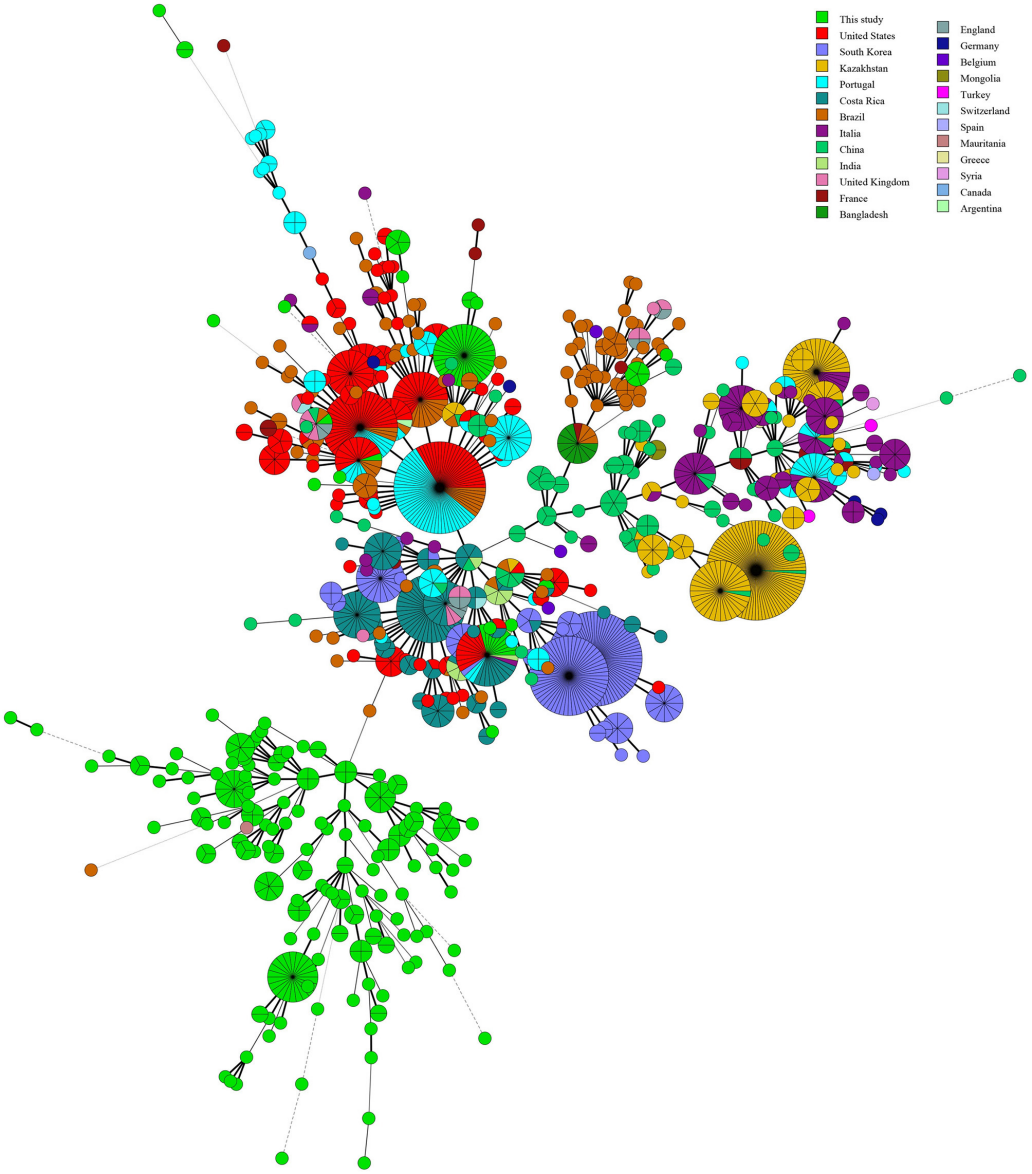
Molecular relationships between *B. abortus* strains from West Africa and worldwide strains based on the MLVA-16 data of 1,476 *B. abortus* strains globally. Color coding according to countries. Circle size indicates the number of strains.

Based on the MST of MLVA-16 data on 309 strains from 17 countries, the strains were divided into two branches (I and II). Branch I comprised strains from Egypt, Zimbabwe, and Uganda (Figure 5), and branch II contained strains from the other 14 countries (Figure 5). Remarkably, only one MVA-16 genotype was shared among two strains from two different countries (Senegal and Nigeria) (Figure 5). Based on the MST of 1,746 strains on a global scale, the majority of strains from this study formed independent clades, and only few strains have shared the same MLVA-16 genotype with strains from many countries, including Brazil, the USA, South Korea, Argentina, India, Italia, Portugal, the UK, Costa Rica, and China (Figure 6).

**Figure 7:**
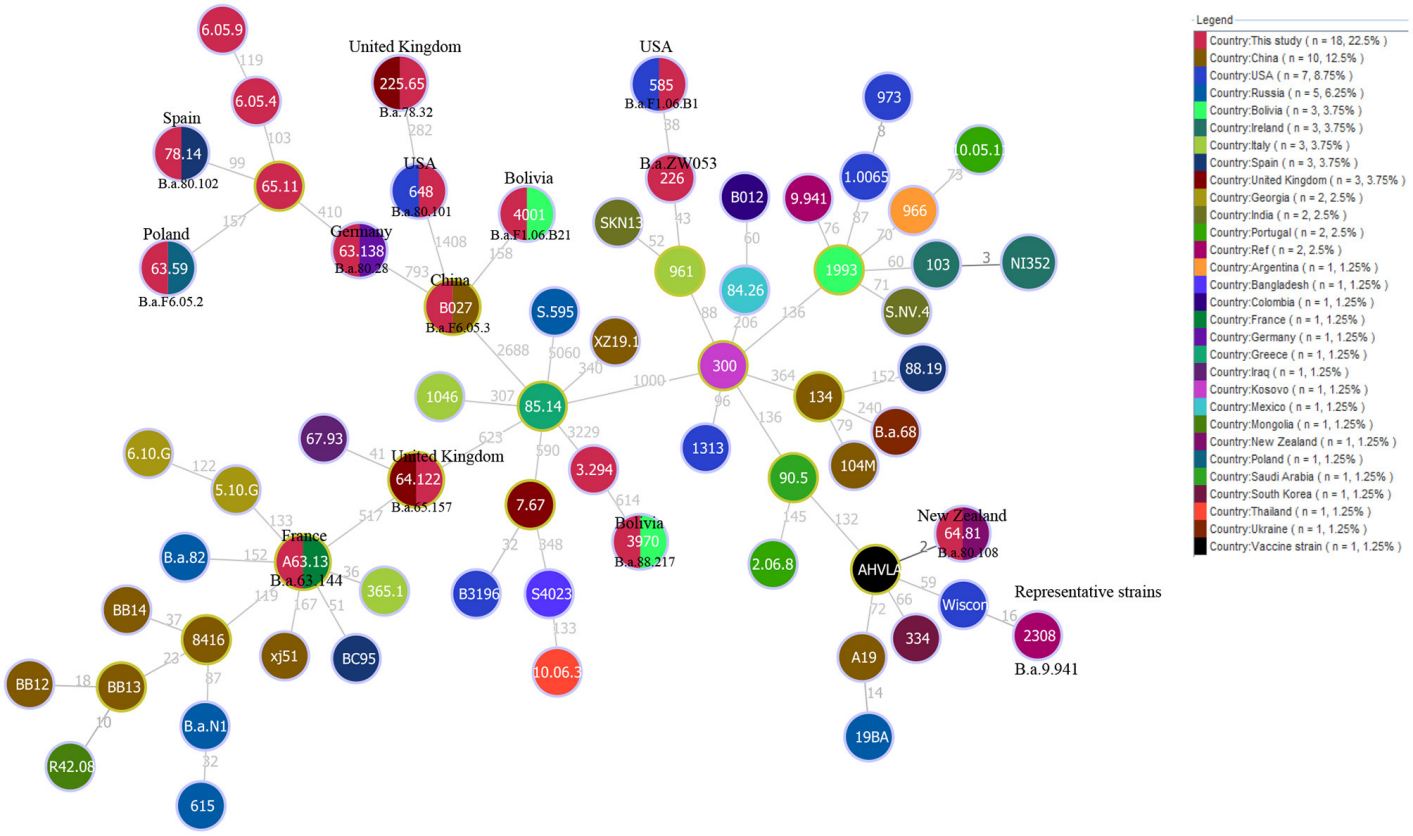
WGS-SNP phylogeny analysis of *B. abortus* strains from the present study and worldwide strains based on the SNP matrix of 80 *B. abortus*. Strains code are marked in black if they share the same SNP genotype with the strain in the circle. The countries marked in the figures indicate that strains from this country have identical SNP genotypes with strains from the present study.

Additionally, the MST based on our WGS-SNP matrix analysis showed that the 18 strains from the present study formed 17 SNP genotypes, and which one was shared by two strains from Mozambique and Zimbabwe (Figure 7). Furthermore, five out of 17 SNP genotypes contained only strains from this study (Figure 7), the remaining 12 were shared SNP genotypes that comprised strains from this study and strains from eight different countries, including the USA (Nigeria and Zimbabwe), the UK (Uganda and Senegal), Germany (Chad), France (Egypt), Poland (Sudan), Spain (Chad), New Zealand (Zimbabwe), Bolivia (Mozambique and Zimbabwe), and China (Sudan) (Figure 7).

## Discussion

In this study, three molecular methods, including MLST, MLVA, and WGS-SNP, were applied to analyze the genetic diversity, population structure, and molecular relationship among 309 *B. abortus* strains from WA with global strains. Our analysis highlights that *B. abortus* strains have a high species/biovars, host diversity, as well as wide geographic distribution in WA. In the present study, more than 90% (280/309) of *B. abortus* strains were isolated from cattle, and the remaining strains were obtained from 10 other hosts, including wildlife. *B. abortus* predominantly infect cattle but also infect other host species and seems to be a spill-over from the dominant host species to other species due to farming and grazing (40), which is also evident from in the present study. A previous study showed that 273 *Brucella* strains from Africa were identified and typed since 1976, among which 272 strains were from cattle and ~95% (260/270) were from native animals showing hygromas (41). Similar when focusing on WA, *B. abortus* strains were historically prevalent mainly in cattle (42–44). Bio-typing identified three biovars in WA, and *B. abortus* bv.3 was the predominant biovar (45).

MLVA11 identified *B. abortus* bv 3 isolated from cattle as new major clusters from WA. In contrast to WA where *B. abortus* biovar 3 strains occur in cattle, *B. abortus* biovar 1 is the dominate biovar in South Africa (46). These data suggest that the predominated *B. abortus* biovars are unique in WA compared to southern Africa, but further investigation is needed to better understand the epidemiological trait of *B. abortus* in Africa.

Cattle production plays a crucial role in Africa including WA, as cattle are traded for status and serve as a “savings account” in nomadic systems in Nigeria (47). It has been reported that 20% of cattle are imported in Nigeria, mostly from Chad and Niger (48). Moreover, according to literature, brucellosis is one of the major transboundary animal diseases in North African countries, while the illegal animal movement was identified as one of the major constraining factors (49).

In cattle, *B. abortus* mainly invades the reproductive organs and causes symptoms such as abortion and a decline in milk productivity, resulting in substantial economic losses (50). Therefore, strengthening disease surveillance in cattle is necessary to map the epidemiology of *B. abortus*. However, strains were found in 10 wildlife species in WA, indicating that the *B. abortus* strains circulate among animal species that are not the preferential hosts. These reservoirs pose a crucial risk to domestic animals and humans. A previous study showed that exploring the wildlife reservoir of brucellosis may represent a new challenge to be faced by the medical and veterinarian community in the twenty-first century (51). Brucellosis in wildlife in this region cannot be neglected, detection and surveillance should be implemented to block the transmission chain.

Although MLVA has a higher resolution power for *Brucella* strains than MLST, both the MLST and MLVA are crucial to epidemiologic surveillance and investigation into the geographical distribution of *B. abortus* strains. MLST analysis highlighted that the predominant circulating *B. abortus* population of WA was native lineages, while three STs were shared by many continents (Figure 2). However, these shared populations were predominant in Europe (ST1 and 2) and America (ST1 and 5), implying that the introduced *B. abortus* lineage was from outside WA, but a further survey into the trade activities of cattle is needed.

The MLVA-11 highlights the high genetic diversity and multiple geographic original of strains from the present study as reflected by the 37 MLVA-11 genotypes were identified among 309 strains that include 22 known and 15 new genotypes. Two predominant MLVA-11 genotypes (317 and 64) were observed in WA (52, 53), while genotype 317 was distributed in Kenya, Senegal, Guinea, Guinea-Bissau, and Rwanda (52, 53), and 64 was also distributed in Senegal and Togo (53). Comparison analysis of global MLVA-11 data suggests that these genotypes originate from WA. Three MLVA-11 genotypes (66, 79, and 82) were shared with strains from Brazil (54), the USA (55), Italy, Portugal (56), France, and South Korea (26). The WA *B. abortus* strains from the present study existed multiple geographic original that suggest introduction from multiple regions outside WA similar to study by Wareth et al. (57).

The global analysis of *B. abortus* strains base on MLVA-16 highlights that the native dominant strains in WA are driving the ongoing prevalence of *B. abortus* in this region. This was similar to findings by Liu et al. (58) suggesting that *B. abortus* strains were transmitted within the national borders of a country despite common geographical origins of the strain in countries along the silk road. *B. abortus* bv 3 strains from Algeria grouped in two separate clusters using MLVA11 which most strains clustering with European isolates from France and Spain while a few strains cluster in African lineage (59). These studies thus indicate presence of introduced strains as well as native strains in WA. Therefore, further extensive bacteriological and molecular investigations are necessary for a comprehensive understanding of the epidemiology of cattle brucellosis in African countries.

WGS-SNP analysis identified *B. abortus* lineages circulating in WA was introduced through the import of animals and/or animal products from multiple countries as 12 of 17 SNP genotypes were from nine countries (regions). The MLST, MLVA, and SNP analyses demonstrated that native strains and introduced lineages are co-driving with the persistence of *B. abortus* circulating in WA. Similarly, WGS-SNP analysis demonstrated that brucellosis in South Africa spreads within the herd on some farms, whereas the introduction of infected animals is the mode of transmission on other farms (33). WGS phylodynamics analysis identified the main *B. abortus* lineage circulate in Costa Rica are widespread while new introductions seem to be more geographically restricted, which might be similar in various other middle- and low-income countries where brucellosis is endemic with similar farming practices and lack of control (60).

Therefore, a control program involving improved surveillance, animal movement restrictions, public health education is suggested. Moreover, there is a strong need for more sustainable molecular data on prevailing *Brucella* strains in WA, and on all susceptible species, including humans, to comprehensively analyze the relatedness between field strains of *Brucella* and the epidemiology of brucellosis within WA countries (29).

## Conclusions

The present study revealed the constant circulation of *B. abortus* strains in cattle throughout WA and neighboring countries. These strains exhibited high species/biovars, host spectrum, and genetic diversity as well as multiple geographic origins. Moreover, most cases were caused by native strains, and few cases resulted from introduced lineages. The surveillance and control of *B. abortus* in WA should be made a priority which can be enhanced by improving molecular databases that can elucidate epidemiological relationships between strains. Regulations should be strictly implemented when introducing animals to prevent the spread of this species (48).

## Data availability statement

The original contributions presented in the study are included in the article/Supplementary material, further inquiries can be directed to the corresponding authors.

## Author contributions

LG and ZLiu performed the data collection and data analysis and drafted the manuscript. MW and QS conducted the data check and critically reviewed the manuscript. ZLiu, ZLi, and XD participated in the design of the study and managed the project. All authors read and approved the final manuscript.

## Supplementary material

Supplementary Figure S1 Genetic relationships based on the STs of *B. abortus* strains (*n* = 129) in this study using eBURST. Re-sampling for bootstrapping = 1,000; minimum number of identical loci for group definition = 3; minimum number of SLVs for subgroup definition = 1. Strains in ST34 are from Sudan (*n* = 24), Nigeria (*n* = 4), and Chad (*n* = 3).

Supplementary Figure S2 Minimal spanning tree (MST) of MLVA-11 genotype diversity based on the multi-loci variable number of tandem repeats (VNTR) 11 data from West African *Brucella abortus* strains. Color coding according to MLVA-11 genotypes. Circle size indicates the number of strains.

Supplementary Table S1 MLST data sets of *B. abortus* strains used in the present study. 129 strains from the present study (Africa) and 631 strains from the outside of Africa.

Supplementary Table S2 The MLVA-16 data sets of *B. abortus* strains used for the present study. 309 marked with gray-used from the present study, MLVA-11 data from the 1,746 *B. abortus* strains used for geographical origin analysis globally and all data were used to determinate the relationship among strains at a global scale.

Supplementary Table S3 A total of 80 *B. abortus* strains genome data from GenBank (*n* = 62) and this study (*n* = 18) were used in the present study for WGS-SNP phylogeny analysis globally.
