# Supplementary figures and images for: Original and introduced lineages co-driving the persistence of Brucella abortus circulating in West Africa
Source: Front Public Health. 2023 Mar 15;11:1106361. doi: 10.3389/fpubh.2023.1106361 (PMC10050740; doi:10.3389/fpubh.2023.1106361)

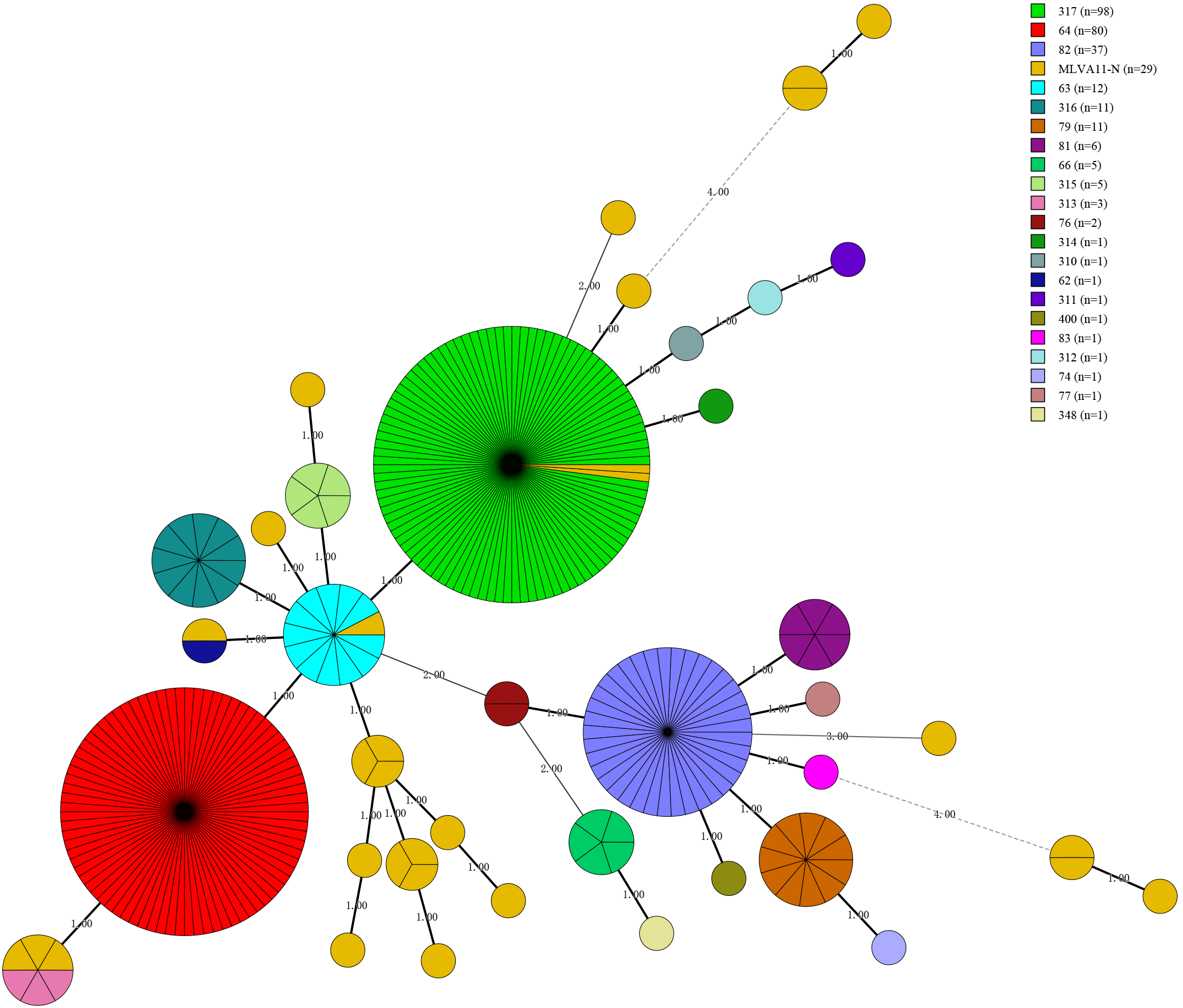

Supplement: Supplementary Figure S1 — Genetic relationships based on the STs of B. abortus strains (n = 129) in this study using eBURST. Re-sampling for bootstrapping = 1,000; minimum number of identical loci for group definition = 3; minimum number of SLVs for subgroup definition = 1. Strains in ST34 are from Sudan (n = 24), Nigeria (n = 4), and Chad (n = 3). [file Image_1.TIF]

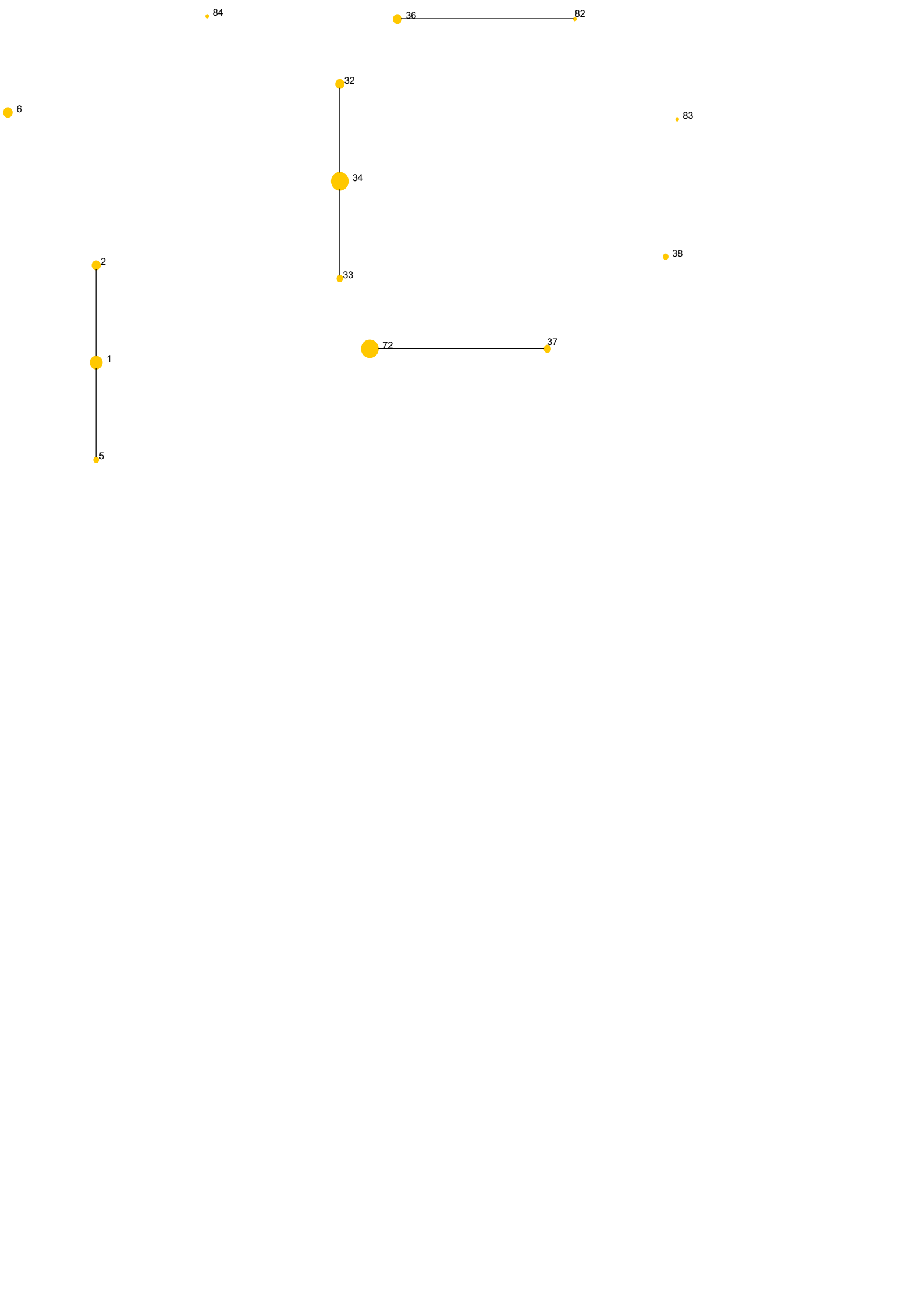

Supplement: Supplementary Figure S2 — Minimal spanning tree (MST) of MLVA-11 genotype diversity based on the multi-loci variable number of tandem repeats (VNTR) 11 data from West African Brucella abortus strains. Color coding according to MLVA-11 genotypes. Circle size indicates the number of strains. [file Image_2.pdf]
